# The Downstream Regulation of Chemokine Receptor Signalling: Implications for Atherosclerosis

**DOI:** 10.1155/2013/459520

**Published:** 2013-04-14

**Authors:** Jyoti Patel, Keith M. Channon, Eileen McNeill

**Affiliations:** ^1^Division of Cardiovascular Medicine, Radcliffe Department of Medicine, University of Oxford, John Radcliffe Hospital, Oxford OX3 9DU, UK; ^2^Division of Cardiovascular Medicine, Radcliffe Department of Medicine, Wellcome Trust Centre for Human Genetics, University of Oxford, Oxford OX3 7BN, UK

## Abstract

Heterotrimeric G-protein-coupled receptors (GPCRs) are key mediators of intracellular signalling, control numerous physiological processes, and are one of the largest class of proteins to be pharmacologically targeted. Chemokine-induced macrophage recruitment into the vascular wall is an early pathological event in the progression of atherosclerosis. Leukocyte activation and chemotaxis during cell recruitment are mediated by chemokine ligation of multiple GPCRs. Regulation of GPCR signalling is critical in limiting vascular inflammation and involves interaction with downstream proteins such as GPCR kinases (GRKs), arrestin proteins and regulator of G-protein signalling (RGS) proteins. These have emerged as new mediators of atherogenesis by functioning in internalisation, desensitisation, and signal termination of chemokine receptors. Targeting chemokine signalling through these proteins may provide new strategies to alter atherosclerotic plaque formation and plaque biology.

## 1. Introduction

GPCRs are a diverse family of seven transmembrane-spanning receptors that activate intracellular signalling pathways by coupling to heterotrimeric G-proteins. They represent one of the largest families of cell-surface receptors with ~1000 encoded by the mammalian genome and are targets for a large number of current therapeutic drugs [1, 2]. GPCRs are activated by a variety of ligands including neurotransmitters, chemokines, hormones, calcium ions, and sensory stimuli. Consequently, they control many physiological processes such as sensory perception, neurotransmission, proliferation, cell survival, and chemotaxis. Given that GPCR signalling is so widespread, and various GPCR subtypes can control different responses; this system requires regulation by processes such as receptor desensitisation, internalisation, and signal termination. In this review, we will give an overview of GPCR activation with the main focus being on the mechanisms of chemokine-mediated GPCR signalling in atherosclerosis. GPCR regulation, and GPCR interacting proteins will be highlighted with examples from experimental models of inflammation providing insights into atherosclerosis.

## 2. Atherosclerosis and Plaque Development

Atherosclerosis is a chronic inflammatory disease of medium to large arteries that is characterised by the accumulation of oxidised low-density lipoprotein (oxLDL) within the arterial wall and a progressive inflammatory cell infiltrate [3, 4]. Monocytes enter at sites of endothelial inflammation and differentiate into macrophages, which accumulate cholesterol to form foam cells [5, 6]. Consequently, fatty streak lesions develop and growth continues into fibrofatty plaques through continued recruitment and differentiation of monocytes and macrophages [5, 6]. T-lymphocytes and vascular smooth muscle cells (VSMCs) migrate to form an intima and a fibrous cap, encasing a core of lipid deposits and a cellular infiltrate of foam cells [7]. A buildup of necrotic cells leads to the formation of an acellular necrotic core which is stabilised by the fibrous cap [8]. Advanced atherosclerotic lesions are further complicated with calcification and degradation of the cap by matrix metalloproteinases (MMPs) which make the plaque vulnerable to rupture [8, 9]. Unstable plaques that rupture release the highly thrombogenic content of the lesion to the circulation and trigger platelet activation and the blood coagulation cascade, which causes thrombus formation at the plaque site [10, 11]. This can lead to vessel occlusion, restriction of blood flow, and subsequently trigger catastrophic clinical events such as myocardial infarction. 

The key role of leukocyte recruitment and its regulation by chemokines has been elegantly demonstrated in experimental models of atherosclerosis. To study the progression of atherosclerosis, gene targeting techniques have created murine models of hyperlipidaemia which have allowed the assessment of disease progression in a time-dependant manner [12]. The apolipoprotein E (ApoE) and LDL receptor (Ldlr) knockout mouse models of atherosclerosis have elevated plasma cholesterol levels when fed a high-fat diet (and on a chow diet in the case of *ApoE* ^−/−^) due to impaired lipoprotein clearance as a result of ligand (ApoE) or receptor (LDLR) deletion [13]. These well-characterised mouse models are predisposed to develop lesions at specific sites throughout the arterial system [14]. As observed in humans, these sites are localised to curved and branched regions of low shear stress at the vessel wall [15, 16], such as the aortic root, the brachiocephalic artery, and the aortic arch. Studies in the *ApoE* ^−/−^ and *Ldlr* ^−/−^ mouse models have given us an insight into the critical cellular processes in plaque formation and progression and identified key molecules in leukocyte recruitment, especially in the chemokine family, using genetic targeting or antagonists of these molecules and their receptors. Increasingly, genetic interventions targeting the pathways downstream of chemokine receptors are also being explored in these and other models of inflammation.

## 3. Chemokines in Atherosclerosis 

The recruitment of inflammatory cells is triggered by the production of chemokines within the plaque microenvironment [3]. Chemokines are a family of small molecular weight proteins of ~8–12 kDa that are divided into four subfamilies based on the position of conserved cysteine residues in their structure (C, CC, CXC, and CX3C) [17, 18]. The major CC and CXC chemokine classes have a highly conserved tertiary structure, characterised by a flexible N-terminus, followed by a cysteine motif, three *β*-sheets, and a C-terminal *α*-helix [19]. The N-terminus, containing a region known as the N-loop, is important in forming chemokine-receptor binding interactions and subsequent activation [20]. Chemokines signal through their G-protein-coupled receptors (GPCRs), and, to date, there are 50 known chemokines and 19 known chemokine receptors, indicating functional redundancy within the chemokine family [21]. Chemokines are able to bind multiple receptors in the same way that an individual chemokine receptor is able to bind multiple chemokines. This compensatory mechanism which results in similar functional responses through different chemokine-receptor pairings underlies the myriad of chemokine-signalling pathways in both homeostasis and inflammation. 

Chemokines are released from endothelial cells, mast cells, platelets, macrophages, and lymphocytes. They are soluble proteins that are produced with a signal peptide which is cleaved before secretion from the cell [17]. Chemokines bind to glycosaminoglycans (GAGs) on the cell surface or in the extracellular matrix (ECM) which serve to immobilise them and promote the formation of a chemokine gradient [22]. In the vasculature, under shear flow conditions, this is necessary for the local concentration of chemokines that are presented on endothelial cells. Chemokines form interactions with corresponding receptors that are highly expressed on leukocytes and are able to direct sequential events in leukocyte trafficking: from bone marrow mobilisation to extravasation from the blood, through to migration into tissues. During inflammation, proinflammatory cytokines (e.g., TNF-*α*, interleukins (IL)-*α*/*β*), bacterial (lipopolysaccharide) and viral (dsRNA) products induce the production of chemokines that then attract leukocytes to the site of inflammation [23]. Controlled leukocyte recruitment is crucial for the generation of an immune response, but inappropriate trafficking can lead to the development of chronic inflammatory diseases.

Chemokines and chemokine receptors have been implicated in atherosclerosis; at the initiation phase of plaque formation during leukocyte adhesion and chemotaxis, during progression and regression. Both CC and CXC chemokines are known to be expressed in murine and human atherosclerotic plaques, and progression correlates with an increased expression of pro-inflammatory chemokines and their receptors within aortas of hyperlipidaemic mice [3]. Studies in mice deficient in a chemokine or chemokine receptor on the ApoE or LDLr knockout background have highlighted the functional role of many chemokines in the recruitment of leukocytes in lesions. For example, mice with deficiency of either CCL2 or CCR2 or with leukocyte CCR2-deficiency on an atherosclerotic background all showed decreased lesion formation in the aortic root [24–27]. In the two former mouse models, this attenuation was accompanied by reduced macrophage numbers in the aortic root. In addition to regulating macrophage migration into plaque, chemokines also control lymphocyte function in atherosclerosis.

Lymphocyte activation occurs during lesion progression, with T-lymphocyte infiltration generally observed in advanced lesions [28]. CD4 and CD8 T-lymphocytes regulate the adaptive immune response through the secretion of TNF-*α* and IFN-*γ* following reactivation by presentation of oxLDL peptide by antigen presenting cells, macrophages, and dendritic cells [29, 30]. *Ccr1* deficiency on the *ApoE* ^−/−^ background has proatherogenic effects, causing an increase in aortic root lesion development and T-lymphocyte infiltration, indicative of a shift to a pro-inflammatory Th1 (T-helper) type response [31]. In contrast, *Ccr5* deficiency on the *ApoE* ^−/−^ background protects mice against diet-induced atherosclerotic lesion formation which is accompanied by the reduced monocyte and T-lymphocyte infiltration in *Ccr5* ^−/−^ aortic roots [31]. The many chemokines and their receptors have been highlighted in experimental models of atherosclerosis which have been extensively reviewed elsewhere [3, 32–34].

In addition to well-defined roles in plaque development, through the regulation of leukocyte recruitment, new roles for chemokines in cell retention and even plaque regression are becoming apparent. The molecules and signals that cause retention of cells within plaque are poorly understood, but there is emerging evidence that chemokines, for example, CX_3_CL1, which serves as a chemoattractant and adhesion molecule for monocytes, and T-lymphocytes is upregulated with human monocyte differentiation into macrophages in response to oxLDL and is required for macrophage/foam cell adhesion to coronary artery smooth muscle cells (SMCs) [35, 36]. This indicates a potential role in macrophage retention in atherosclerotic plaques.

Recent models of atherosclerosis regression when plaque regression is initiated by the normalisation of hyperlipidaemia with surgical transfer of plaque to wild type animals or by genetic intervention, chemokines may control the efflux of lipid laden macrophages [37]. In the regression environment, macrophages have been shown to exhibit a dendritic like state and emigrate to draining lymph nodes in a CCR7-dependent manner [37]. 

From these studies, it is clear that chemokine signalling plays an important function in leukocyte trafficking in the pathogenesis of atherosclerosis. Despite greater than 20 years of research on chemokines and chemokine receptors in atherosclerosis, no drugs have yet been licenced. Although compounds such as MLN-120, an anti-CCR2 monoclonal antibody are in development, there are comparatively few chemokine inhibitors given the vast number multiple chemokine-chemokine receptor pairings [38]. Identifying the mechanisms that regulate the downstream chemokine receptor-signalling pathways would reveal potential therapeutic targets in atherosclerosis.

## 4. GPCR Activation and Signal Transduction

The general paradigm for GPCR activation is that binding of agonists to extracellular domains of the receptor induces conformational changes in the seven transmembrane spanning domain. This facilitates interactions with intracellular heterotrimeric G-proteins and enables transmission of the signal. Heterotrimeric G-proteins are composed of *α*, *β*, and *γ* subunits. 

**Figure 1:**
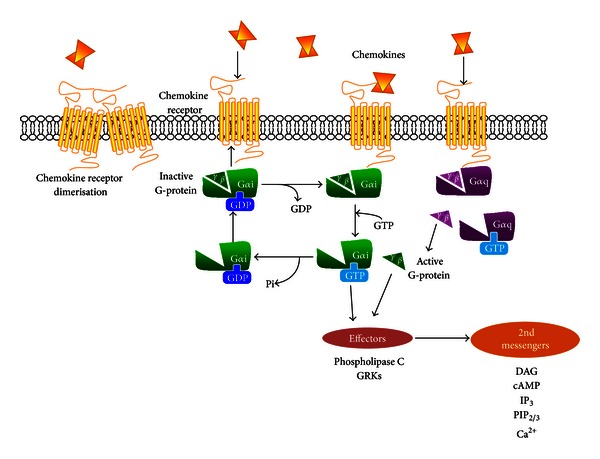
Schematic summary of chemokine-mediated GPCR signalling. Chemokine binding to the receptor induces conformational changes in its transmembrane domain to allow it to couple to a heterotrimeric G-protein. Chemokine receptors predominantly couple to G*α*i proteins (green) but also G*α*q proteins (purple). Chemokines also interact with their receptors to induce the formation of receptor dimers or oligomers. This induces GDP to GTP exchange at the nucleotide binding site of the G*α* subunit. This causes the dissociation of the GTP-bound G*α* subunit from the G*βγ* heterodimers and the activation of downstream signalling effectors. This leads to the production of second messengers which further propagate signal transduction pathways that cause a cellular response. Inactivation of the G-protein occurs through hydrolysis of GTP, allowing the G*α*-GDP to recombine with the *βγ* dimers.

Upon activation, GPCRs act as guanine nucleotide exchange factors (GEFs) for the G*α* subunit which results in guanosine diphosphate (GDP) to guanosine triphosphate (GTP) exchange [1]. This leads to the dissociation of the GTP-bound G*α* subunit from the G*βγ* heterodimers, thus allowing both subunits to propagate downstream signal transduction pathways (Figure 1). There are 23 known mammalian G*α* proteins divided into four broad subfamilies: G*α*s, G*α*i/o, G*α*q/11, and G*α*12/13. The majority of chemokines mediate their signals via G*α*i proteins, although several have been postulated to interact with alternative G*α* proteins such as G*α*q [39, 40].

## 5. Chemokine-Mediated GPCR Signalling 

Chemokine-stimulated GPCRs can initiate several downstream effectors that ultimately lead to actin polarisation, shape change, and directed cell movement. Stimulation of G*α*i subunits can result in the activation of calcium channels and inhibition of adenyl cyclases and cyclic adenosine monophosphate (cAMP) production [41]. However, it is the G*βγ* subunits, which are required for chemotaxis [42]. The activation of these subunits can trigger a number of signalling effectors such as GPCR kinases (GRKs), ion channels, and phospholipase C-*β* (PLC-*β*) [41]. PLC-*β* catalyses phosphatidylinositol (3,4,5)-trisphosphate (PIP_3_) to inositol trisphosphate (IP_3_) and diacylglycerol (DAG). IP_3_ causes a release in calcium from endoplasmic reticulum (ER) stores, and DAG can activate protein kinase C (PKC), which is involved in receptor regulation through phosphorylation and desensitisation. Moreover, both G*α* and G*βγ* subunits can activate phosphoinositide 3-kinase (PI3K) independently that results in the activation of the kinases, Akt and the mitogen-activated proteins kinases (MAPKs) [43]. 

PI3K phosphorylates phosphatidylinositol (4,5)-bisphosphate (PIP_2_) to PIP_3_ at the cell membrane [44, 45]. An increase in PIP_3_ results in the localised recruitment of signalling proteins containing PIP3-pleckstrin homology (PH) domains [44]. These proteins then drive actin polymerisation and morphological changes at the leading edge of the cell, causing it to polarise and move forward towards the highest concentration of chemokine [44, 46]. Given the widespread action of GPCRs, it is crucial that GPCR expression, activation, and signalling are tightly controlled by cellular regulatory mechanisms. 

## 6. Regulation of GPCRs

### 6.1. Regulation of GPCRs: Internalisation

GPCR signalling can be regulated at the level of receptor expression by the process of internalisation which aims to reduce the amount of available GPCRs on the cell surface (downregulation), thus attenuating receptor-mediated signalling. Following ligand activation, intracellular domains of receptors are phosphorylated by kinases such as the second messenger kinases and GRKs. This targets them for internalisation into the cell via endosomes for lysosomal degradation [47]. In addition to degradation, in some cases, endocytosed receptors are dephosphorylated by endosomal-associated phosphorylases (resensitisation) and recycled back to the cell surface [1, 48]. 

Chemokines are able to influence chemokine receptor internalisation and recycling. CCR7 is efficiently internalised when engaged by CCL19 in comparison to its other ligand CCL21, as CCL19 induces greater phosphorylation of the receptor [49, 50]. For the recruitment of arrestin proteins which are described later, phosphorylation is a prerequisite [49]. Internalisation of CCR7/CCL19 is arrestin-dependent mechanism but not for CCR7/CCL21 [49]. However, internalisation of a receptor is not required for the migration of a cell, as reported in studies by Hsu et al. on wild type, and phosphorylation deficient N-formyl peptide receptors that fail to be internalised in U937 myeloid cells have no defects in chemotaxis to formyl-methionyl leucyl phenylalanine [49, 51]. Differential effects on receptor recycling can also modulate GPCR signalling and the magnitude of the cellular response through one or more selective chemokine receptors. CCL5 causes CCR1, CCR3, and CCR5 internalisation but through different pathways, such that CCR3 is partially degraded and recycled whereas CCR5 is completely recycled in eosinophils [52, 53]. The implications for *in vivo* trafficking are unclear but as well as causing chemotaxis, chemokines may induce retention through hyporesponsiveness. If cells migrate to an inflammatory site and become localised, they may not be responsive to other chemokines that signal through the same receptor and stop movement until they encounter another chemokine signal [52].

Intriguingly, some receptors have the ability to continue to signal or initiate other signal transduction pathways during endosomal trafficking [48]. This can have confounding effects through excessive signalling and promote inflammatory disease. Truncated CXCR4 variants implicated in the immunodeficiency WHIM (Warts, Hypogammaglobulinemia, Infections, and Myelokathexis) syndrome, are able to mediate chemotactic responses but unable to be desensitised and internalised [54]. This mechanism is believed to alter the function of leukocytes in WHIM, since the receptors activate G-proteins effectively that result in enhanced CXCL12-induced chemotaxis [54, 55]. This appears to result in the abnormal retention of neutrophils in the bone marrow. Additionally, CXCR4 is a good example of how regulation of GPCR signalling can be influenced by receptor dimerisation. The formation of heterodimers between wild-type and truncated CXCR4 is thought to account for the increased signalling to CXCL12 activation and their inability to be endocytosed [54]. In addition to internalisation, receptor dimerisation/oligomerisation can influence agonist affinity, potency, and receptor phosphorylation and may therefore regulate functional responses of the GPCR [56, 57]. Chemokine receptor dimers and oligomers have been identified in both the CC and CXC subfamilies [58]. Many of these studies have been performed in cell lines expressing CCR2 and/or CCR5 and assessed ligand binding in cells expressing CCR2/5 heterodimers [59]. Chemokine receptor homodimerisation results in G*α*i-mediated signalling (Figure 1), whereas chemokine receptor heterodimerisation is induced by the presence of two different chemokines and activates G*α*q signalling [60]. This can cause downstream activation of different G-protein effectors.

**Figure 2:**
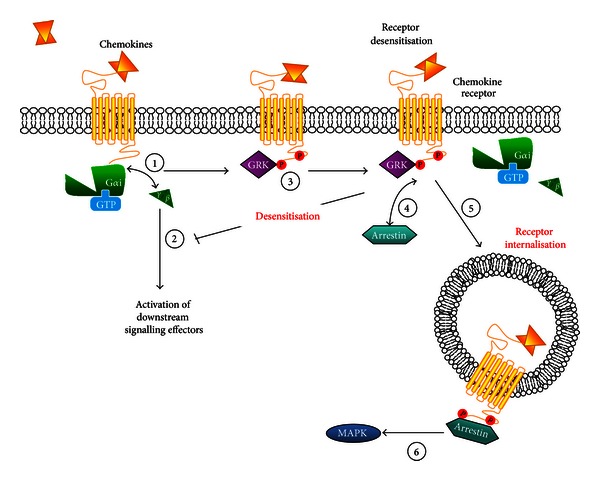
GRKs and arrestins in chemokine-mediated GPCR signalling. Following receptor activation and G-protein dissociation (1) downstream signalling pathways are activated (2). Ligand-activated GPCRs are phosphorylated by GRKs (3), resulting in the recruitment of arrestins (4). This uncouples the receptor from its G-protein, thereby attenuating further receptor signalling. The binding of arrestins to the receptor promotes internalisation of the receptor (5) that can result in the down-regulation of receptor, but can also contribute to a second round of signalling such as activation of the MAPK cascade (6).

**Figure 3:**
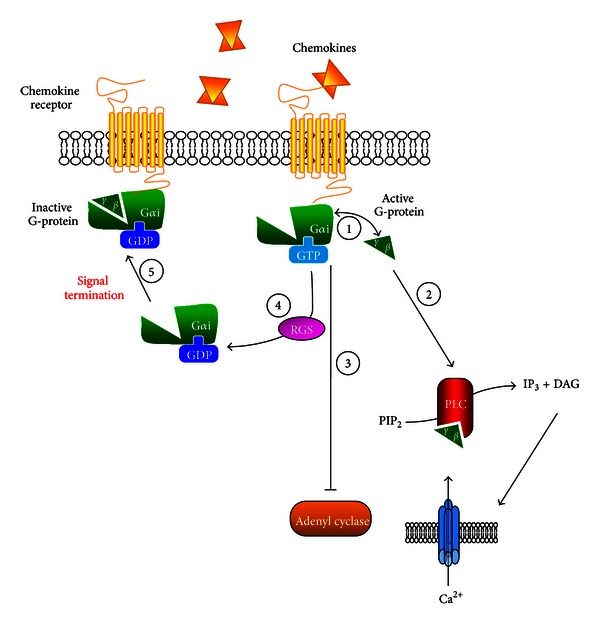
RGS proteins in chemokine-mediated GPCR signalling. Following receptor activation and G-protein coupling, the G*α* and G*βγ* subunits dissociate (1) and regulate downstream effector functions such as activation of PLC (2) and inhibition of adenyl cyclase (3). The *βγ* subunit activates PLC, leading to local production of IP3 and DAG from PIP2. IP3 activates calcium channels which results in a release of calcium. RGS proteins enhance the GTPase activity of the G*α*-subunit, controlling the rate of GTP hydrolysis (4). This promotes the formation of the inactive G-protein and subsequently terminates chemokine signalling (5).

The rapid process of endocytosis and downregulation of GPCRs is closely related to the process of desensitisation which regulates GPCRs at the functional level, since they are both augmented by the interactions between intracellular domains of the GPCR and heterotrimeric G-protein and cytoplasmic proteins, such as GRKs, arrestins, and RGS proteins. The processes behind chemokine-mediated GPCR internalisation, desensitisation, and termination are highlighted in Figures 2 and 3.

### 6.2. Arrestins

Arrestin proteins act as adapters at GPCRs following receptor activation and phosphorylation, and function in desensitisation through two processes. Firstly, binding of arrestins to the phosphorylated receptor sterically blocks receptor and G-protein interaction [61]. Secondly, arrestin binding targets the GPCR to clathrin coated pits at the cell surface resulting in subsequent receptor internalisation to endosomes for degradation, dephosphorylation, and recycling back to the membrane [62, 63] (Figure 2). **β*-arrestin 1* gene expression is upregulated in splenocytes and mesenteric lymph nodes following induction of the inflammatory disease, adjuvant arthritis in rats [64]. In knockout studies, **β*-arrestin 2*-deficient neutrophils exhibit increased calcium signalling and GTPase activity, accompanied by reduced CXCR2 receptor internalisation in response to CXCL1 [65]. The recruitment of neutrophils was increased in response to CXCL1 in the air pouch model to assess *in vivo* chemotaxis [65]. **β*-arrestin 2*-deficient T-lymphocytes have decreased CXCR4/CXCL12 mediated migration *in vitro*; confirming that T-lymphocyte chemotaxis has a critical role in lung inflammation *in vivo* [66]. *In vivo* studies have demonstrated that *β*-arrestin 2 is involved in allergic asthma, since allergen-treated knockout mice have no accumulation of Th2 cells in lungs that is characteristic of inflammation in asthma [66]. 

Arrestins have functions independent of receptor desensitisation. Arrestins are multifunctional proteins that connect different signalling effectors in cells, in particular, the MAPK system. **β*-arrestin 2* overexpression in human embryonic kidney (HEK)-293 and HeLa cells enhances *in vitro* migration to CXCL12 by augmenting p38 MAPK activation [67]. This mechanism behind p38 MAPK function in chemotaxis remains unclear but is likely to occur through the phosphorylation of an F-actin cap binding protein [67]. Together, these studies imply a more complex role of arrestins in different aspects of chemokine signalling and leukocyte recruitment and both protective and nonprotective roles in disease. In cardiovascular inflammation, **β*-arrestin 2* levels are increased in human atherosclerotic arteries in comparison to non-atherosclerotic arteries [68]. Animal studies in the LDLr knockout model have shown that *β*-arrestin 2 is proatherogenic [69]. **β*-arrestin *2^−/−^ *Ldlr* ^−/−^ mice develop less atherosclerosis in the aorta after 12 weeks on a western type diet that is linked to a reduced SMC content in aortic root lesions. SMC proliferation and migration into the arterial intima is linked to the development of atherosclerosis suggesting that arrestins regulate this process.

### 6.3. Regulation of GPCRs: Desensitisation

Desensitisation is a regulatory mechanism in controlling receptor activity, attenuating signalling to prolonged or repeated stimulation [70]. Two types of desensitisation exist: homologous and heterologous. Homologous desensitisation occurs when an agonist that is specific for a receptor causes loss of a response and is a result of phosphorylation of the receptor by GRKs and subsequent *β*-arrestin action. Much evidence comes from *in vitro* systems, where high concentrations of ligand are required for homologous desensitisation and whether this occurs *in vivo* remains to be determined. In contrast, heterologous desensitisation refers to the activation of one receptor in causing desensitisation of multiple receptors in their active or inactive forms by kinases such as protein kinase A (PKA) and protein kinase C (PKC) that are stimulated by second messengers [62, 71]. PKA and PKC directly uncouple GPCRs from G-proteins by phosphorylation of intracellular serine and threonine residues in the intracellular loop and the carboxy terminus of the GPCR [47]. The importance of GRKs and *β*-arrestins in chemokine mediated responses have been demonstrated in knockout and overexpressing mice where GPCR phosphorylation, desensitisation, and internalisation are affected. The regulatory proteins discussed in this review are summarised in Table 1 in the context of atherosclerosis. Receptor desensitisation is an important feedback mechanism preventing acute or chronic receptor overstimulation that could lead to abnormal cellular signalling.

### 6.4. GRKs

GRKs phosphorylate agonist occupied GPCRs, which increases the affinity of the receptor for arrestin-dependent binding [61] (Figure 2). This results in receptor and G-protein uncoupling and receptor internalisation [62]. In contrast to second messenger-dependent protein kinases, much higher concentrations of agonist are required to phosphorylate and desensitise receptors by this pathway [62]. Several GRKs show high expression in immune cells (GRK2, -3, -5, -6) and their expression levels are regulated in inflammation [2]. *In vitro* studies with proinflammatory cytokines IL-6 and IFN-*γ* induce GRK2 protein downregulation in human peripheral blood mononuclear cells (PBMCs) and GRK2 and GRK6 levels are reduced in PBMCs from patients with rheumatoid arthritis (RA) and in splenocytes from experimental mouse models of multiple sclerosis (MS) [2]. *GRK*2^+/−^ T-lymphocytes have increased migration to the chemokines CCL3 and CCL4 [77]. This suggests that during inflammation when there is an increase in pro-inflammatory mediators; this is likely to cause an downregulation of GRK2/5/6 activity *in vivo* [61]. In non-pathological inflammation, this is required for a controlled response to chemokine stimulation, but in chronic inflammation, this may lead to enhanced chemokine signalling and increased cell infiltration to an inflammatory site.

In contrast, enhanced GRK activity has been associated with cardiovascular disorders including hypertension and cardiac hypertrophy. An upregulation of *GRK2* in nonmyocyte cardiac cells is linked to enhanced *β*1-adrenergic receptor signalling and is associated with heart failure [78]. *GRK*6^−/−^ neutrophils show enhanced calcium signalling and chemotactic responses to leukotriene B4 (LTB4) and CXCL12 *in vitro* [79, 80]. *GRK*2^+/−^ mice develop acute onset of experimental autoimmune encephalomyelitis (EAE) that is accompanied by significantly increased cellular infiltration in the spinal cord [81]. Homozygous *GRK2* ^−/−^ mice are embryonically lethal [78]. *GRK2* ^+/−^ T-lymphocytes display increased calcium mobilisation, migration, and downstream Akt and extracellular signal-regulated kinase (Erk1/2) signalling to CCR5 ligands [77]. 

Based on these findings, it might be expected that targeted *GRK*2^+/−^ deletion would lead to enhanced chemokine signalling which would propagate inflammatory cell recruitment *in vivo* and augment atherosclerotic lesion formation in *GRK*2^+/−^ mice with hyperlipidaemia. Contrary to these findings, atherosclerosis is attenuated in mice with a haemopoietic deficiency in *GRK2* ^+/−^, that is accompanied by a 79% decrease in necrotic core size [72]. Interestingly, macrophage content in the lesions from *GRK2* ^+/−^ mice was significantly greater than control mice as indicated by % MOMA-2 positive staining. However, a conditional GRK2 deficiency in a macrophage/granulocyte specific transgenic model (*LysM-Cre GRK2* ^flox/flox^) did not display any differences in atherosclerosis indicating that macrophages were not solely responsible for the phenotype observed in *GRK*2^+/−^ bone marrow chimeras [72]. Furthermore, circulating monocytes were reduced in *GRK*2^+/−^ mice, highlighting a potential role for GRK2 in monocyte mobilisation and may account for the increased plaque macrophage content observed in *GRK*2^+/−^ mice.

In contrast to GRK2, GRK5 activity is antiatherogenic, since *GRK5* ^−/−^ *ApoE* ^−/−^ mice have an increase in lesion area in comparison to *ApoE* ^−/−^ mice through two different cell-type regulatory mechanisms in monocyte/macrophages and SMCs [73]. In SMCs, GRK5 is able to promote the degradation of the non-GPCR proatherogenic receptor, platelet-derived growth factor receptor-*β* (PDGFR*β*) in lysosomes which is thought to reduce PDGF-mediated SMC proliferation and migration [73]. GRK5 regulates monocyte chemotaxis; *in vitro GRK5* ^−/−^ monocytes have increased migration to CCL2, a ligand for the GPCR, CCR2 and colony stimulating factor-1, a ligand for the CSFR-1, a receptor tyrosine kinase [73]. CCL2-mediated leukocyte migration is instrumental in atherosclerotic lesion progression and responsible for the increased macrophage content in lesions from *GRK5* ^−/−^ mice. These findings highlight the potential mechanisms in both monocyte retention and emigration after their migration across the endothelium and present new strategies to limit atherosclerotic lesion progression.

### 6.5. Regulation of GPCRs: Signal Termination

GPCR signalling can be terminated by desensitisation followed by internalisation. However, further regulation leading to signal termination can be achieved through G-protein interaction with RGS proteins. In the last ten years, there has been growing evidence indicating the importance of RGS proteins in contributing to signal termination, without interacting with the receptor itself, but by action at the G*α*-subunit coupled to the receptor. Chemokine receptors couple to G*α*i subunits that are present in leukocytes, with G*α*i2 and G*α*i3 being principally expressed in murine lymphocytes and macrophages [2, 82]. Regulation of G*α*i-signalling pathways *in vivo* is required for proper functioning of the immune system, for correct homing of cells to lymphoid organs and for cell trafficking to sites of inflammation [83]. Inactivation of G*α*-subunits is driven by a number of processes such as the intrinsic GTPase activity of the G*α* protein that hydrolyses GTP to GDP, enabling the heterotrimer to reform [41]. This process can be accelerated by RGS proteins that act as GTPase activating proteins (GAPs) and thus promote G-protein inactivation by downregulating the intracellular response to repeated ligand stimulation [84] (Figure 3).

Currently, there are 30 known mammalian RGS protein family members, divided into eight subfamilies that are based on sequence homologies [85]. Subfamily proteins contain a conserved ~120 amino acid residue RGS domain or an RGS-like domain; however, homologous regions outside this domain are thought to be shared within each subfamily. RGS proteins exert GTPase activation via binding of the RGS domain to the GTP-binding domain of the G*α* protein. The G*α* active site is composed of three switch regions that undergo conformational changes during activation and deactivation from GTP to GDP bound states [84]. The RGS domain stabilises the transition state of these switch regions of the G*α* subunit upon binding but does not make contact with bound GTP and therefore is not directly involved in catalysis [84, 86]. By altering the conformation of the active G*α*-GTP complex, RGS proteins are able to accelerate the rate of GTP hydrolysis by the GTPase, by as much as >2000 fold *in vitro* [84]. Given the widespread expression of RGS proteins in several tissues and the numerous cellular functions mediated by GPCRs, RGS proteins have been identified to have a critical role in signalling pathways involved in cardiovascular, phototransduction and CNS functions. Given the potency of RGS proteins in modulating GPCR function, dysregulation of RGS proteins may lead to pathological disorders such as atherosclerosis. Currently, there is limited published work on the role of RGS proteins in inflammatory disease, but we will discuss emerging data on RGS control of leukocyte function that provide insights for atherosclerosis.

In the cardiovascular system, RGS2 and RGS5 control physiological regulatory responses to blood pressure and cardiac rhythmicity, whereas changes in the expression of *Rgs3* and *Rgs4* have been associated with heart failure in humans [87]. RGS1 has been linked to cardiovascular disorders and in particular chemokine signalling in inflammation. *Rgs1* mRNA has been reported to be expressed in the left ventricular myocardium of patients with dilated and ischemic cardiomyopathy [88], and mRNA transcripts are also present in the heart and aorta of septic animals [89]. 

Emerging evidence for a role for RGS1 specifically in atherosclerosis is provided by several human studies that have measured *Rgs1* gene expression in vascular disease. During inflammation, increased chemokine signalling can contribute to disease progression through overactivation and recruitment of monocyte-macrophages.

Anger et al. found *Rgs1* mRNA upregulation in advanced calcified aortic valve stenosis [90]. In a gene array study to investigate plaque rupture, stable and unstable human carotid artery atherosclerotic plaques were examined. *Rgs1* mRNA was found to be upregulated 12-fold with plaque instability [74]. Likewise, gene expression profiling of human atherosclerotic and nonatherosclerotic coronary arteries, also measured an increased *Rgs1* expression in atherosclerotic coronary arteries [76]. Studies from our laboratory have identified *Rgs1* as one of the differentially expressed genes in the thoracic aortas of 16-week-old atherosclerotic *ApoE* ^−/−^ mice, compared with 8-week-old *ApoE* ^−/−^ mice (unpublished data). *Rgs1* expression is associated with atherosclerotic plaque progression, and furthermore these results correlated with the expression of the macrophage marker, CD68 indicating a role for *Rgs1* in macrophage function.

The role of RGS1 in the regulation of *in vivo* chemotactic responses has been highlighted in studies of *Rgs*1^−/−^ mice. These studies have been limited to lymphocytes and the control of B-lymphocyte homing to lymph nodes, since RGS1 is highly expressed in germinal centres [91]. However this provides an insight into its potential role in leukocyte function. The migration of activated B-lymphocytes to these centres is regulated by their expression of distinct chemokine receptors such as CXCR4 and CXCR5 [92]. Additionally *in vitro*, *Rgs*1^−/−^ B-lymphocytes show increased chemotaxis and calcium mobilisation to CXCL12 and CXCL13 [91]. Following chemokine pre-exposure, they still retain this exaggerated response to these chemokines due to impaired desensitisation [82, 91]. These differences support altered *in vivo* function as *Rgs*1^−/−^ mice exhibit excessive germinal centre formation following immunisation and abnormal trafficking of antibody-secreting cells, implying inappropriate recruitment of B-lymphocytes into germinal centres during the humoral immune response [91]. Collectively, these studies present evidence that RGS1 is key regulator of leukocyte trafficking and is critical in downregulating the response to sustained chemokine signalling. 

Further recent evidence for a role for RGS1 in leukocyte chemotaxis is more complex. In assessing *in vivo* migration, Agenès et al. used parabiotic mice to investigate naïve and regulatory T-lymphocyte (Treg) migration [93]. Naïve T-lymphocytes migrated more readily than Tregs. Chemotaxis of naïve T-lymphocytes was correlated with a downregulation of RGS1, whereas Tregs were characterised by an elevated expression of RGS1. This suggested that an increase in RGS1 may increase desensitisation and reduce the capacity of T-lymphocytes to migrate [93]. A recent study has shown that RGS1 expression is higher in human gut T-lymphocytes in comparison to peripheral blood T-lymphocytes and that it reduces intestinal T-lymphocyte migration to lymphoid homing chemokines [94]. Furthermore, when *Rgs*1^−/−^ and wild type T-lymphocytes were transferred in the colitis model in *Rag2* deficient mice which lack mature lymphocytes, *Rgs1* deficiency showed a protective phenotype indicating RGS1 in having a potential role in T-lymphocyte retention in the gut [94]. A proatherogenic role exists for T-lymphocytes, since a deficiency in this cell type inhibits atherosclerotic lesion development [95]. These studies in lymphocytes would give us an insight into the role of RGS1 in atherosclerosis. Targeted *Rgs1* deletion may lead to enhanced chemokine signalling in macrophages due to a lack of desensitisation resulting in increased chemotaxis. This may propagate inflammatory cell recruitment *in vivo* which will augment atherosclerotic lesion formation in *Rgs1* ^−/−^ mice on an atherosclerotic background.

## 7. Conclusions

Many of the *in vivo* studies highlighted here have demonstrated the importance of regulatory proteins in chemokine biology and that dysregulation of GPCR signalling can lead to both pro- and antiatherogenic responses. This underlines that enhanced or impaired desensitisation and that signal termination of GPCRs can lead to altered leukocyte trafficking in inflammation. Their roles in controlling leukocyte recruitment may yield insight into the mechanism of pathological recruitment and retention of leukocytes at sites of atherosclerotic plaque. Understanding this additional layer of control and specificity to our understanding of disease biology may help us both further understand the specificity that is achieved by this widely expressed system and allow us to target this system with therapeutics. 

To target these downstream regulatory pathways, we need to understand when inhibition or enhancement of activity is required, given the opposing roles of the regulatory proteins in atherosclerotic mice. Heterozygotic *GRK2* ^+/−^ mice on the LDLr background have reduced atherosclerosis [72], and overexpression of *GRK2* is linked to heart failure [96], implying that inhibiting GRK2 would be beneficial. Currently, the GRK2/3 family have been targeted for inhibition, but this has proven ineffective due to inhibitors lacking selectivity [96]. In contrast, in neurological disorders, gene therapy has been raised as a potential tool for enhancing GRK6 activity by overexpression [96]. GRK5 would be an ideal target to be therapeutically activated since *GRK*5^−/−^ *ApoE* ^−/−^ mice have increased atherosclerosis [73]. However, enhancing activity of GRK5/6 requires further elucidation on the mechanism behind their cellular concentrations, degradation and transcription, and is currently speculative. 

Modulating chemokine signalling by targeting RGS proteins is still in early development and requires much more understanding on their physiological regulation. Current research has focussed on altering RGS protein interactions with G*α* protein subunits or by changing the localisation or expression of a particular RGS protein in a cell type [97]. Given the regulation of RGS protein expression in different cells and that they act at different G*α* proteins, it may be possible to achieve a high degree of target specificity. For example, suppressing RGS1 in inflammatory tissue by an inhibitor which might result in enhanced G*α*i signalling might prevent the retention of cells which would normally progress inflammation. Different RGS proteins would require either inhibitors or potentiators to attenuate or enhance G-protein action, and the possibilities have been discussed in detail by Zhong and Neubig [98]. In comparison to chemokine biology, the role of GPCR regulatory proteins in atherosclerosis is still limited, but with the development of new experimental mouse models in the last 5 years, this field will expand and enable the discovery of novel therapeutic strategies in cardiovascular inflammation. 

## Figures and Tables

**Table 1:** GPCR signalling regulatory proteins in atherosclerosis.

Regulatory protein	Experimental evidence in atherosclerosis	Reference
*β*-arrestin 2	(i) **β*-arrestin 2 * ^−/−^ *Ldlr * ^−/−^ mice have reduced aortic atherosclerosis (ii) **β*-arrestin 2 * ^−/−^ *Ldlr * ^−/−^ mice have reduced SMC content in the aortic root	[69]

GRK2	(i) *GRK2 * ^+/−^ *Ldlr * ^−/−^ chimeric mice have reduced atherosclerosis and necrotic core in the aortic root (ii) *GRK2 * ^+/−^ *Ldlr * ^−/−^ chimeric mice have increased macrophage and VSMC content in aortic root lesions (iii) *CCL5*-induced *in vivo* migration of leukocytes is increased *GRK2 * ^+/−^ *Ldlr * ^−/−^ chimeras	[72]

GRK5	(i) *GRK5 * ^−/−^ *ApoE * ^−/−^ mice have increased atherosclerosis in aorta than *ApoE * ^−/−^ mice (ii) *GRK5 * ^−/−^ *ApoE * ^−/−^ mice have increased macrophage and VSMC proliferation in aortic root lesions (iii) *GRK5 * ^−/−^ monocytes have increased migration to atherogenic stimuli *in vitro *	[73]

RGS1	(i) *Rgs1* expression is upregulated in thoracic aortas from *ApoE* ^−/−^ mice at 16 weeks of age in comparison to *ApoE* ^−/−^ mice at 8 weeks of age and wild-type C57BL/6J mice (ii) *Rgs1* was found to be upregulated in unstable segments of plaque from human carotid endarterectomy specimens over stable segments from the same patient (iii) *Rgs1* upregulated in human atherosclerotic coronary arteries	Unpublished data, Channon laboratory [74] [68]

RGS2	(i) Genetic polymorphisms in *Rgs2* have been associated with intima-media thickening of the carotid artery in patients with hypertension	[75]

RGS5	(i) *Rgs5* expression is downregulated in SMCs of atherosclerotic plaques from nonhuman primates	[76]
